# Inadvisably presenting APACHE scores as parametric data: a study of 200 original articles from leading journals

**DOI:** 10.1186/cc9922

**Published:** 2011-03-11

**Authors:** R Kam, C Bunce, JM Handy

**Affiliations:** 1 Imperial College London, UK; 2 Moorfields Eye Hospital NHS Foundation Trust, London, UK; 3 Chelsea & Westminster Hospital, Imperial College London, UK

## Introduction

The APACHE score, used to indicate severity of systemic illness in patients, is the sum of separate points given for different aspects of organ dysfunction. It is therefore ordinal data, and in intensive care patients should not simply be assumed to be well approximated by a normal distribution. This study aimed to discover what proportion of recent intensive care literature is presenting this score inadvisably as normal data.

## Methods

Twenty of the most recent original articles containing 'APACHE' or 'Acute Physiology and Chronic Health Evaluation' were identified from each search engine of 10 highly cited journals with notable intensive care literature content. Studies presenting an average score and a measure of central spread were included. Statistical methods used were recorded.

## Results

Approximately 70% of identified papers presented APACHE data as means and standard deviations, and 48% used these data in parametric tests. Eighty-one per cent did not mention assessment of skewness or kurtosis and only 7% documented the test used to assess whether the distribution appeared normal.

## Conclusions

Inadvisable presentation and processing of APACHE data is commonplace in critical care journals and authors should exercise greater awareness of the potentially skewed distribution of the data. Medians and interquartile ranges suit its ordinal nature better. Subjecting APACHE data to parametric analysis when non-normally distributed will increase the risk of type 1 or type 2 errors depending on the nature of departure from non-normality.

